# Control of Podocyte and Glomerular Capillary Wall Structure and Elasticity by WNK1 Kinase

**DOI:** 10.3389/fcell.2020.618898

**Published:** 2021-02-02

**Authors:** Zhenan Liu, Joonho Yoon, Chonlarat Wichaidit, Ankita B. Jaykumar, Hashem A. Dbouk, Addie E. Embry, Liping Liu, Joel M. Henderson, Audrey N. Chang, Melanie H. Cobb, Richard Tyler Miller

**Affiliations:** ^1^Department of Internal Medicine, Division of Nephrology, University of Texas Southwestern Medical Center, Dallas, TX, United States; ^2^Medicine Service, VA North Texas Health Care System, Dallas, TX, United States; ^3^Department of Pharmacology, University of Texas Southwestern Medical Center, Dallas, TX, United States; ^4^Department of Pathology and Laboratory Medicine, Boston University School of Medicine, Boston, MA, United States

**Keywords:** glomerulus, podocyte, WNK1 kinase, elasticity, cytoskeleton

## Abstract

Cytoskeletal structure and its regulation are essential for maintenance of the differentiated state of specific types of cells and their adaptation to physiologic and pathophysiologic conditions. Renal glomerular capillaries, composed of podocytes, endothelial cells, and the glomerular basement membrane, have distinct structural and biophysical properties and are the site of injury in many glomerular diseases. Calcineurin inhibitors, immunosuppressant drugs used for organ transplantation and auto-immune diseases, can protect podocytes and glomerular capillaries from injury by preserving podocyte cytoskeletal structure. These drugs cause complications including hypertension and hyperkalemia which are mediated by WNK (With No Lysine) kinases as well as vasculopathy with glomerulopathy. WNK kinases and their target kinases oxidative stress-responsive kinase 1 (OSR1) and SPS1-related proline/alanine-rich kinase (SPAK) have fundamental roles in angiogenesis and are activated by calcineurin inhibitors, but the actions of these agents on kidney vasculature, and glomerular capillaries are not fully understood. We investigated WNK1 expression in cultured podocytes and isolated mouse glomerular capillaries to determine if WNK1 contributes to calcineurin inhibitor-induced preservation of podocyte and glomerular structure. WNK1 and OSR1/SPAK are expressed in podocytes, and in a pattern similar to podocyte synaptopodin in glomerular capillaries. Calcineurin inhibitors increased active OSR1/SPAK in glomerular capillaries, the Young’s modulus (E) of glomeruli, and the F/G actin ratio, effects all blocked by WNK inhibition. In glomeruli, WNK inhibition caused reduced and irregular synaptopodin-staining, abnormal capillary and foot process structures, and increased deformability. In cultured podocytes, FK506 activated OSR1/SPAK, increased lamellipodia, accelerated cell migration, and promoted traction force. These actions of FK506 were reduced by depletion of WNK1. Collectively, these results demonstrate the importance of WNK1 in regulation of the podocyte actin cytoskeleton, biophysical properties of glomerular capillaries, and slit diaphragm structure, all of which are essential to normal kidney function.

## Introduction

Characteristics of the cell cytoskeleton are basic determinants of the structural and biophysical properties of cells and ultimately tissues. In the kidney, renal glomerular capillaries function at relatively high pulsatile pressures and filter blood to form the initial urine. They consist of fenestrated endothelial cells that participate in filtration, podocytes, or visceral epithelial cells that have several orders of branched processes, and a glomerular basement membrane (GBM) that is synthesized by both endothelial cells and podocytes. The primary and secondary processes of podocytes wrap around the capillaries. The terminal foot processes, the interdigitating final extensions of neighboring podocytes, anchor these cells to the GBM. These podocyte processes also provide structural and mechanical support to glomerular capillaries by opposing and accommodating hemodynamic force. The narrow gap, or filtration slit, between adjacent terminal foot processes is bridged by specialized tight junctions, known as slit diaphragms that constitute the final component of the filtration barrier. Glomerular disease is frequently associated with podocyte injury and actin cytoskeletal remodeling which disrupt interactions with neighboring podocytes and the GBM. This podocyte failure is characterized by foot process effacement, broadening and flattening of foot processes, and loss of slit diaphragms. The result is abnormal filtration function as well as disrupted podocyte and capillary wall mechanical properties, culminating in detachment of podocytes and their loss in the urine.

The With No Lysine (WNK) kinases are a family of serine-threonine kinases with the unusual location of a conserved lysine residue in the active site (Xu et al., 2000). These kinases have been studied most in their roles in epithelial transport because mutations in two of them, WNK1 and WNK4, are associated with renal transport phenotypes. The WNKs control vectorial transport in epithelial cells and cell volume in non-epithelial cells where they regulate cation-chloride cotransporters (Kochl et al., 2016; Shekarabi et al., 2017). WNKs are activated by hypotonic and hypertonic medium and osmotic shrinkage of cells. With an inhibitory Cl binding site in the active site of the kinase domain, WNKs function as Cl sensors (Zagorska et al., 2007; Piala et al., 2014; Chen et al., 2019). The adaptor KLHL3 binds WNKs to promote their degradation by Cullin3-mediated ubiquitination (Ohta et al., 2013; Cornelius et al., 2018). Many actions of WNK kinases are carried out by their substrates, the Sterile 20 Kinases, oxidative stress-responsive kinase 1 (OSR1) and its close homolog SPS1-related proline/alanine-rich kinase (SPAK).

With No Lysine 1 is ubiquitously expressed in non-renal and renal cells, including podocytes, while WNK4 is predominantly expressed in renal tubules (Shekarabi et al., 2017). WNK1 participates in fundamental biologic processes including angiogenesis, and mitosis (Xie et al., 2009, 2013; Tu et al., 2011; Gallolu Kankanamalage et al., 2016). WNK1 kinase is also involved in cell migration and adhesion where it controls cytoskeletal structure and migration-associated local cell volume fluctuations as demonstrated in endothelial cells, T cells, corneal epithelial cells and certain cancers, notably glioblastoma cells (Haas et al., 2011; Dbouk et al., 2014; Zhu et al., 2014; Kochl et al., 2016; Desjardins et al., 2019).

Tacrolimus (FK506) and cyclosporine A (CsA) are immunosuppressive drugs used following organ transplantation to prevent rejection and to treat autoimmune diseases. FK506 and CsA act by inhibiting the calcium and calmodulin-dependent phosphatase calcineurin. Among other substrates, calcineurin dephosphorylates KLHL3 leading to increased degradation of WNK1 and WNK4 (Ohta et al., 2013; Shekarabi et al., 2017; Ishizawa et al., 2019). As a consequence of increased WNK expression, FK506 and CsA cause increased activity of the WNK targets OSR1/SPAK explaining in part the volume-dependent hypertension and hyperkalemia seen with their use (Hoorn et al., 2011). Calcineurin inhibitors also have protective effects on podocytes in glomerular injury models, including preservation of cytoskeletal structure, and can improve the course of some human renal diseases (Meyrier, 2005; Charbit et al., 2007; Faul et al., 2008; Plank et al., 2008; Bensman and Niaudet, 2010; Li et al., 2015; Shen et al., 2016). However, use of these drugs can be limited by nephrotoxicity, characterized by vasculopathy, glomerular injury, tubular atrophy, interstitial fibrosis, hypertension, and hyperkalemia (Issa et al., 2013).

Despite the importance of WNK1 kinase signaling in vascular tissue, and the toxic and beneficial effects of calcineurin inhibitors in the kidney, the roles of WNK kinases in these processes are not fully understood. We studied the responses of isolated glomeruli and cultured podocytes to calcineurin inhibitors and WNK1 inhibition to investigate the contributions of WNK1 to the structural and mechanical properties of glomerular capillaries and podocytes.

## Experimental Procedures

### Reagents

Antibodies recognizing the indicated proteins were from the following sources: WNK1 (Origene 06363PU-N), OSR1 was from MyBiosSource, and pOSR1(Ser325)/SPAK(Ser373) – Millipore-Sigma, anti-synaptopodin – Santa Cruz; β-actin – Sigma; Cortactin H222 Cell Signaling; FK506 and cyclosporin A – LC Laboratories, sorbitol – Sigma, and Jasplakinolide and Latrunculin B – Fisher; WNK463 – MedChemExpress. All fluorescent antibodies were purchased from Invitrogen.

### Cell Culture and Lysis Conditions

Conditionally-immortalized mouse podocytes were cultured in RPMI 1640 with 10% FBS as previously described (Tandon et al., 2007). Cells proliferated at 33°C and differentiated at 37°C with addition of IFNγ. Confluent cells in RPMI 1640 without FBS were treated with 0.5 μM FK-506, 1 μM CsA, 0.5 M sorbitol, 0.5 M NaCl, and 0.5 to 20 μM WNK463 for the times shown, then lysed in 1× RIPA (Cell Signaling) with Halt phosphatase/protease inhibitor cocktail (Thermo) containing 1-3% NaF and Trisodium tetraoxovanadate. DMSO (final concentration 0.05% for added chemicals) was used as a control. A range of WNK463 concentrations (1 to 20 μM) were used to confirm minimal cytotoxicity (Supplementary Figure 1). Reversibility of WNK463 treatments were evaluated by washout studies at the highest dose (Supplementary Figure 2).

### Isolation of Mouse Glomeruli

To isolate glomeruli, kidneys were removed from mice, placed in iced buffer, decapsulated, and dissected on ice to isolate the cortices from the medullae. The cortices were minced with a razor blade and pushed through a screen (180 μm, W.S. Tyler Co, Cleveland, OH, United States) with a flexible metal spatula. The minced tissue was suspended in PBS with 5.5 mM glucose and 0.3 mM pyruvate (GIBCO), filtered through a 90 μm nylon mesh (Falcon) to remove vessels and large pieces of tissue. The filtrate was collected in a 45 μm mesh nylon filter (Falcon) and contained intact glomeruli with minimal contamination (<2%) by tubular cells (Schlondorff, 1990; Embry et al., 2016, 2018). Glomeruli were maintained in DMEM with 0.1% FBS at room temperature and treated with drugs (FK506 5 μm, CsA 10 μM, and WNK463 0.5–20 μM) as indicated for 2 h before elasticity measurements or confocal imaging.

Animal research was performed in accordance with the UT Southwestern Medical Center Animal IACUC guidelines. The research study protocol (number 2014–0078) was approved by the UT Southwestern Medical Center Animal IACUC (NIH OLAW Assurance Number A3472-01). UT Southwestern Medical Center is fully accredited by the American Association for the Assessment and Accreditation of Laboratory Care, International (AAALAC). Animals are housed and maintained in accordance with the applicable portions of the Animal Welfare Act and the Guide for the Care and Use of Laboratory Animals. Veterinary care is under the direction of a full-time veterinarian boarded by the American College of Laboratory Animal Medicine. Mice were sacrificed for experiments by first anesthetizing with Avertin and then euthanizing by cervical dislocation.

### Measurement of Glomerular Elasticity

The elastic moduli of glomeruli isolated from 3–6 month old mice were measured using a microprobe indenter device (Levental et al., 2010; Embry et al., 2016, 2018). Briefly, a tensiometer probe (Kibron, Inc, Helsinki, Finland) with a 250 μm radius flat-bottom needle was mounted on a 3-D micromanipulator with 160 nm step size (Eppendorf, Inc) attached to a Leica inverted microscope. A glass slide containing a dilute sample of glomeruli was imaged by bright field illumination and the bottom of the probe was brought through the air-water interface until it was just above the surface of a single glomerulus of diameter approximately 60 μm. The probe was calibrated using the known surface tension of a pure water/air interface, and the stress applied to the probe as it was lowered onto the glomerulus was measured as a function of indentation depth. In principle, this deformation geometry is that of an infinite plane compressing a spherical object. The absolute values of elastic modulus can be calculated from appropriate models that require assumptions about the adherence of the glomerulus to the probe and the glass slide, whether the glomerular elasticity is modeled as a uniform sphere or an elastic shell, and other structural factors that confound calculation of the magnitude of elastic modulus from the force-indentation data alone. In all cases indentations were kept below 15 μm to avoid large strains that could damage the glomeruli. After the largest indentations, measurements were repeated at small strains to confirm that the deformations were recoverable.

### Fractionation of F/G-Actin (Glomeruli)

Isolation of filamentous (F) and globular (G) actin was performed as previously described (Wyss et al., 2011; Embry et al., 2016). Briefly, glomeruli were treated with DMSO (0.05%), FK-506 (5 μM), Jasplakinolide (10 μM), or Latrunculin B (1 μM) for 2 h, then lysed in 20 mM Hepes pH = 7.4, 100 mM NaCl, 1 μM ATP, 1 mM NaVO_4_, 50 mM NaF, 1% Triton X-100, and protease inhibitor mix as above. Lysates were passed 6 times through a 25-gage needle and then centrifuged at 100,000 × *g* in a TAL-100 rotor for 1 h at 4°C. The supernatant was removed for G-actin analysis, while the pellet containing F-actin was resuspended in 15 mM Hepes pH 7.5, 150 μM NaCl, 1% Triton X-100, 1% Na-deoxycholate, 0.1% SDS, 10 mM EDTA, 1 mM dithiothreitol, 1 mM NaVO_4_, and protease inhibitor mix. Samples were mixed 1:1 with 2x Laemmli sample buffer, and proteins were separated on 10% acrylamide gels in SDS.

### Fractionation of F/G Actin (Cultured Podocytes)

For cell fractionation of F/G-actin, Podocytes were grown to 80–90% confluence in culture medium containing 10% FBS, then changed to serum-free medium for 1 h. Cells were then treated with serum-free medium containing DMSO, FK506 (0.5 μM), Jasplakinolide (1 μM), or Latrunculin B (4 μM) for 3 h. Cells were lysed in F-actin stabilization buffer [G/F KIT Cat. #BK037 CYTOSKELETON, 50 mM PIPES pH 6.9, 50 mM NaCl, 5 mM MgCl2, 5 mM EGTA, 5% (v/v) Glycerol, 0.1% Non-idet P40, 0.1% Triton X-100, 0.1% Tween 20, 0.1% 2-mercapto-ethanol] at 37°C for 30 min. Lysate debris was pre-cleared by centrifugation at 350 × *g* for 5 min at room temperature, then separated into supernatant (S) and pellet (P) fractions by centrifugation at 100,000 × *g* at 37°C for 1 h. F-actin containing pellets were resolublized in 100 μl of F-actin depolymerization buffer (8 M Urea, 10% Glycerol, 20% SDS, 1 M DTT, and 1.5 M Tris pH6.8) on ice for 1 h, pipetting up and down several times every 15 min. Equivalent volumes of input, supernatant, and pellet fractions were analyzed by SDS-PAGE and western blot.

### Three-Dimensional Collagen Matrix Contraction Assay

Methods for preparing cell-containing collagen matrices have been described previously (Liu et al., 2014; Embry et al., 2018). Briefly, collagen matrices containing cultured podocytes (2 × 10^5^ cells/200 μL gel) were polymerized for 1 h at 37°C. The gels were released from their substrates to float in RPMI 1640 medium with or without FK506, WNK463, 10% FBS, or DMSO (control) for 4 h and then fixed with 4% paraformaldehyde/PBS. Images of collagen matrices were obtained with an Epson photo scanner. Gel area was analyzed using ImageJ, and reduction in area was calculated as: original area (12 mm diameter circle) – area after treatment. Relative gel area reduction was normalized to the untreated (DMSO) control condition. Statistical analyses were performed using GraphPad Prism software.

## Wound-Induced Cell Migration

Equal numbers of podocytes were seeded on collagen-coated six well plates. Confluent cell monolayers were washed and scratch wounds were created with a 1 ml pipette tip. Culture medium (10% FBS or serum-free) was replaced with medium containing DMSO, FK506 (0.5 μM), or FK506 (0.5 μM) + WNK463 (2 μM). Images were obtained immediately after wound creation and before cell migration. Plates were returned to the incubator and cells were allowed to migrate for 18 h, at which time repeat images were obtained.

## Immunofluorescence Staining

Treated glomeruli were spun down at 5,000 *g* for 5 min, resuspended and fixed in 4% paraformaldehyde for 30 min, and then washed 3x in PBS. Washed glomeruli were resuspended in PBS, 100 μl pipetted onto a slide, and attached to a coverslip overnight at 4°C. The slide was rinsed in PBS to remove unattached glomeruli and further fixed with 4% paraformaldehyde for 1 h at room temperature. Attached and fixed glomeruli on coverslips were then washed 3x in PBS, blocked with SEA BLOCK Blocking Buffer (PIERCE) for 40 min, then permeabilized with 0.5% Triton X-100 in PBS for 10 min. Glomeruli on coverslips were then stained using standard procedures, and imaging was performed using a Zeiss LSM 880 confocal microscope.

## Microscopy and Image Analysis

Confocal imaging was performed in the Cell Biology and Imaging Core in the O’Brien Kidney Research Core, on a Zeiss LSM880 with Airyscan laser scanning microscope equipped with Plan-Apochromat 10x/0.3 NA, 20x/0.8 NA, 25x/0.8 NA, and 63x/1.40 NA oil-immersion objective (ZEISS, Oberkochen, Germany). Fluorescence images were acquired using ZEN black 2.3 software with a 20x/0.8 NA or 63x/1.40 NA objective and Zeiss Immersion Oil 518F was used for the 63x/1.40 NA objective. Experiments were performed at constant room temperature. Images or regions of interest (ROIs) were further processed with ZEN 2.6 (blue edition) software.

## Image Analysis

Podocytes were seeded at low density in 2 of 96 well imaging plates (BD Falcon 353219), treated with the following conditions: control, FK506, WNK463, and FK506 + WNK463 – with and without serum (FBS), and then stained with Hoechst, Rhodamine Phalloidin (542/565), and pOSR1/SPAK (Alexa 488) as detailed above. Images were taken (25 frames per well) using an INCell Analyzer 6000 (GE). CellProfiler was used to identify the nucleus and cell boundary from Hoechst and Phalloidin, respectively, (Kamentsky et al., 2011). Cell size and number of “touching” neighbors for all the cells were obtained. We utilized a MeasureObjectIntensity Distribution module to (1) equally divide each cell along its radius from the center of its nucleus to the membrane edge into 6 bins, (2) measure pOSR1/SPAK average intensities from all the bins in each cell. The average pOSR1/SPAK intensity of outer most bin, i.e., the 6th bin was then used to represent the cell membrane/peri-membrane area. Cells with poorly and under-segmented boundaries were excluded by setting a threshold of cell area/nuclear area ≤ 2. Furthermore, cells with two or more touching neighboring cells were excluded (Note: on average, about 30–40% of cells were excluded). Ruffling cells (cells with lamellipodia) usually have “high” pOSR1/SPAK intensities at the membrane regions. Cells with average intensity in the 6th bin equals or higher than 90th percentile were considered to be “ruffling.” The range was carefully chosen by visual inspection. There were 12 replicate wells for each condition (from 2 plates) and roughly 100–150 cells per replicate well.

## Electron Microscopy

Tissue samples were fixed with 2.5% (v/v) glutaraldehyde in 0.1 M sodium cacodylate buffer (pH7.4). After three rinses with 0.1 M sodium cacodylate buffer, samples were embedded in 3% agarose and sliced into small blocks (1 mm^3^), rinsed with the same buffer three times and post-fixed with 1% osmium tetroxide and 0.8% potassium ferricyanide in 0.1 M sodium cacodylate buffer for one and a half hours at room temperature. Samples were rinsed with water and then block stained with 4% uranyl acetate in 50% ethanol for 2 h. They were then dehydrated with increasing concentration of ethanol, transitioned into propylene oxide, infiltrated with Embed-812 resin and polymerized in a 60°C oven overnight. Blocks were sectioned with a diamond knife (Diatome) on a Leica Ultracut 7 ultramicrotome (Leica Microsystems) and collected onto copper grids, post stained with 2% aqueous uranyl acetate and lead citrate. Images were acquired on a JEM-1400 Plus transmission electron microscope equipped with a LaB_6_ source operated at 120 kV using an AMT-BioSprint 16 M CCD camera.

### Statistical Analysis

Student *t* test and one-way analysis of variance were used to determine statistical significance.

## Results

### WNK1 and OSR1/SPAK Are Expressed in Podocytes

**FIGURE 1:**
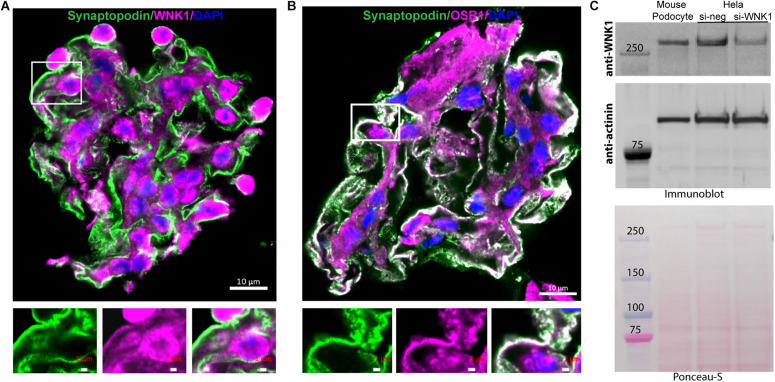
WNK1 and OSR1 are expressed in glomerular podocytes. **(A)** Localization of WNK1 (magenta) and **(B)** OSR1 (magenta) with synaptopodin, a podocyte marker (green) in mouse glomeruli using confocal microscopy. A selected region of the merged double-stained image is enlarged to show overlap (white) of the kinases with synaptopodin below each panel. The images show that WNK1 and OSR1 overlap with synaptopodin indicating that all three proteins are expressed in the cytoplasm of glomerular podocytes. Scale bar in the mages of glomeruli is 10 μm, and in the magnified images is 1 μm. **(C)** Expression of WNK1 in cultured mouse podocytes and specificity of antibody for WNK1. Mouse podocyte cell lysates were separated on a 3–8% Tris-acetate gel and immunoblotted for WNK1. As cultured mouse podocytes are not easily transfected using standard methods, human Hela cells were transfected with siRNA toward WNK1 (si-WNK1) or scrambled control (si-neg), and lysates were used to demonstrate specificity of the antibody. Actinin was used as the immunoblot loading control, and the Ponceau-S stained membrane confirms even transfer of proteins. Molecular mass of protein standards is indicated. Relative to control, siWNK reduced WNK protein by 70 ± 8%.

Renal glomeruli are vascular structures responsible for producing the initial urinary filtrate. Glomerular podocytes are the cells that support glomerular capillaries and form the filtration barrier. Because of the importance of WNK protein kinases in the kidney and the vasculature, we investigated the contributions of WNKs to glomerular structure and function. Single cell RNAseq data from the Humphreys laboratory at Washington University^1^ shows that WNK1 mRNA is expressed at moderate levels in podocytes, mesangial cells and endothelial cells. Expression of mRNAs for WNK2, WNK3, and WNK4 is negligible or low in these cells, which suggests minimal contributions of these other family members to glomerular processes. Based on these data suggesting that WNK1 is the predominant WNK family member in glomeruli and podocytes, we examined expression of WNK1 in freshly isolated mouse glomeruli using confocal microscopy. WNK1 (Figure 1A) and its closely related substrate kinase OSR1 (Figure 1B) are expressed in a pattern similar to synaptopodin, a podocyte marker (Figure 1A). Synaptopodin (green) is located in capillary loops in the podocyte cytoplasm. WNK1 and OSR staining (magenta) are also observed in the cytoplasm of podocytes. The enlarged images below Figures 1A,B show capillary walls where overlap of synaptopodin (green) and WNK1 or OSR1 (magenta) is seen as white. Western blots of cultured mouse podocyte extracts show expression of WNK1 (Figure 1C). The specificity of the antibody used was confirmed by siRNA-mediated depletion of WNK1 from Hela cells.

### Calcineurin Inhibitors Increase WNK Kinase Activity in Cultured Podocytes

**FIGURE 2:**
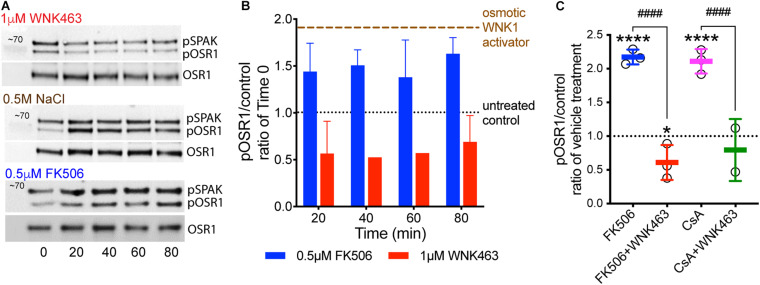
Calcineurin inhibitor-induced increases in pOSR1/SPAK are inhibited by the WNK inhibitor WNK463. **(A)** Representative immunoblots of pOSR1/SPAK and total OSR1 as a loading control in podocyte lysates from cells treated with 1 μM WNK463, 0.5 M NaCl, or 0.5 μM FK506 at indicated times. **(B)** Time course of OSR1 phosphorylation in cultured podocytes in response to FK506 (blue), WNK463 (red) relative to basal phosphorylation at time 0 (black). Phosphorylated OSR1 in response to hypertonic extracellular conditions with added osmotic WNK activators, 0.5 M NaCl or 0.5 M sorbitol (brown tracing) are shown as positive control. The averaged pOSR1/SPAK values ± S.D. were: osmotic activators- 1.8 ± 0.3, *N* = 10; FK506- 1.6 ± 0.3, *N* = 14; and WNK463- 0.6 ± 0.3, *N* = 9. **(C)** Increased phosphorylation of OSR1/SPAK by FK506 (0.5 μM, blue) or cyclosporine A (1 μM, pink), is inhibited by WNK463 (1 μM) at 60 min with indicated treatments. The fold pOSR1/SPAK accumulation at 60 min in CsA-treated podocytes (1 μM) was nearly identical to that in FK506 treated podocytes (2.2 ± 0.11 for FK506 and 2.1 ± 0.25 for CsA), In both conditions pOSR1/SPAK was reduced by 1 μM WNK463 (FK506 0.62 ± 0.25, and CsA 0.79 ± 0.46). Relative amounts of pOSR1 increased by calcineurin inhibitors is significantly inhibited by WNK463. *p < 0.05 vs control, ****p < 0.001 vs control, ^####^*p* < 0.001 FK506 or CsA vs FK605 or CsA + WNK463 by ANOVA.

To measure the effects of calcineurin inhibitors on WNK kinase activity in podocytes, cells were treated with 0.5 μM FK506, and WNK activity was measured as accumulation of pOSR1/SPAK (pSer 325) over time. As positive controls, WNK was activated with 0.5 M NaCl or 0.5 M sorbitol. The panWNK inhibitor WNK463 was used to demonstrate a requirement for WNK in OSR1/SPAK activation (Figure 2; Yamada et al., 2016a, b). WNK463 reduced phosphorylation of both SPAK and OSR1 below that of untreated podocytes indicating that OSR1/SPAK are partially activated by WNK prior to osmotic stimulation (Figures 2A,B). Both 0.5 M NaCl and 0.5 M sorbitol increased pOSR1/SPAK by nearly 2-fold within 20 min and phosphorylation was sustained for over 1 h (Figure 2B, brown tracing at top). FK506 also increased pOSR1/SPAK by 20 min (blue bars), with no additional accumulation by 80 min. Inhibition of WNK kinase activity with WNK463 reduced pOSR1/SPAK to half of their activity in untreated cells. We compared the effects of FK506 and CsA on podocyte pOSR1/SPAK and found, as expected, that both calcineurin inhibitors increase WNK activity as determined by increased pOSR1/SPAK sensitive to WNK463 (Figure 2C). The extent of pOSR1/SPAK accumulation at 60 min in CsA-treated podocytes was nearly identical to that in FK506-treated podocytes, and in both cases, pOSR1/SPAK was reduced to values below basal by 1 μM WNK463.

### FK506 Increases Glomerular Elastic Modulus (E) by a WNK-Dependent Mechanism

**FIGURE 3:**
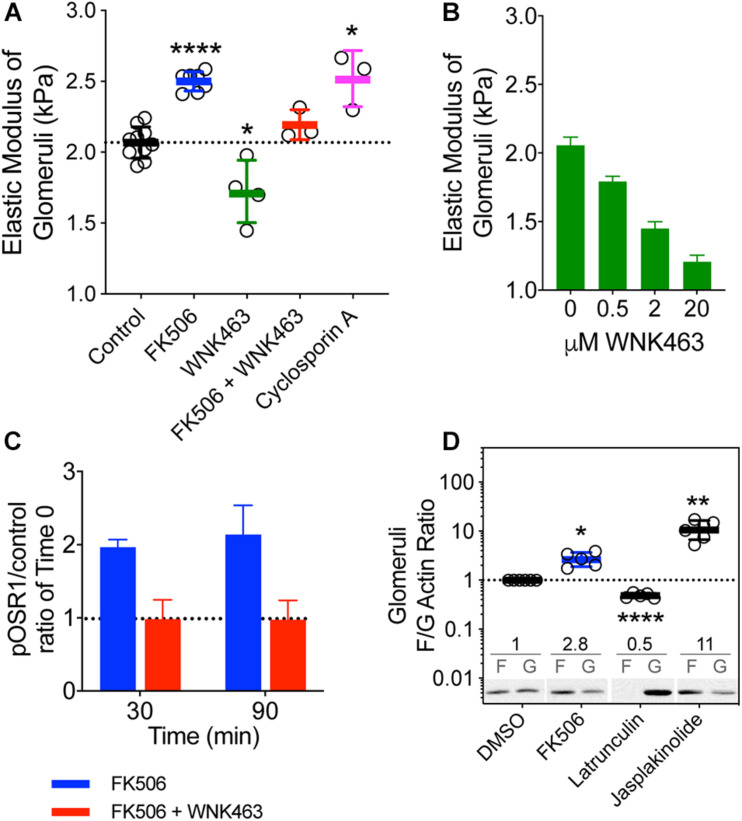
Effects of calcineurin inhibition on the biophysical properties of mouse glomeruli. **(A)** Mouse kidney glomerular elastic modulus (E, Young’s modulus) response to FK506 (5 μM), cyclosporine A (10 μM), and WNK463 (10 μM). FK506-induced increase in E is attenuated by WNK kinase inhibition with WNK463. The E of normal control glomeruli from 3–6 month-old mice is 2.01 ± 0.03 kPa. FK506 (0.5 μM) increased E to 2.50 ± 0.03 kPa. WNK463 (2 μM) decreased E to 1.7 ± 0.2 kPa. WNK463 blocked the FK506-induced stiffening of the glomeruli, reducing the value to 1.97 ± 0.03 kPa, indistinguishable from the control value of 2 kPa. CsA increased E to 2.52 ± 0.11 kPa. ****p < 0.0001 and *p < 0.05 by ANOVA. Mouse glomeruli were freshly isolated and incubated in equivalent volumes of DMSO (Control) FK506, CsA, or WNK463, for 2 h. Glomerular elasticity was measured by microindentation as described in the methods section. **(B)** Increasing doses of WNK463 caused progressive decreases in E. Average values (kPa) ± S.E. are as follows: Control- 2.1 ± 0.59, *N* = 2; 0.5 μM – 1.8 ± 0.4, *N* = 6; 2 μM – 1.5 ± 0.5, *N* = 5; 20 μM – 1.2 ± 0.5, *N* = 5. *N* refers to the number of glomeruli measured. **(C)** FK506 (0.5 μM) increases glomerular pOSR1/SPAK over basal control levels at 30 and 90 min and the response is inhibited by 10 μM WNK463. *p < 0.05, **p < 0.01, by ANOVA **(D)** Relative to vehicle control (DMSO), FK506 (0.5 μM, blue) increases glomerular F/G actin ratios by 2.8 ± 0.5-fold (*N* = 6), Latrunculin B (1 μM) 0.48 ± 0.05-fold (*N* = 5), and Jasplakinolide (10 μM) 11.4 ± 4.5-fold. Average F/G actin ratios are indicated above representative immunoblot images for each treatment. Values shown are mean ± SD.

To determine if calcineurin inhibitors affect glomerular elasticity through effects on WNK kinases, we isolated mouse glomeruli and used microindentation to measure Young’s modulus (E) following treatment with FK506, CsA, or WNK463 (Figure 3A; Levental et al., 2010; Embry et al., 2018). The E of untreated glomeruli from 3–6 month-old mice was 2.0 kPa. FK506 increased E by 25%, to 2.5 kPa, and 2 μm WNK463 reduced E to 1.7 kPa. WNK463 blocked the FK506-induced stiffening of the glomeruli, reducing E to values comparable to controls. The effects of CsA on glomerular elasticity were comparable to those of FK506. WNK463 reduced E of glomerular capillaries in a dose-dependent manner from 2.1 (C) to 1.2 kPa over a dose range of 0.5 to 20 μm (Figure 3B). As was the case in cultured podocytes, FK506 increased pOSR1/SPAK approximately two-fold in isolated glomeruli at 30 and 90 min, and the effect was inhibited by WNK463 (Figure 3C). These results indicate that effects of FK506 on glomerular stiffening require WNK kinase activity. We assessed the effect of FK506 on the structure of the podocyte actin cytoskeleton by measuring the F/G actin ratio in glomeruli treated with FK506. For comparison latrunculin was used to depolymerize actin and jasplakinolide was used to increase actin polymerization (Figure 3D). FK506 increased the F/G actin ratio by 2.8-fold, consistent with the contribution of increased F-actin to the glomerular stiffening response to FK506 and CsA.

### FK506-Increased F-Actin and Podocyte Traction Force Require WNK Kinase Activity

**FIGURE 4:**
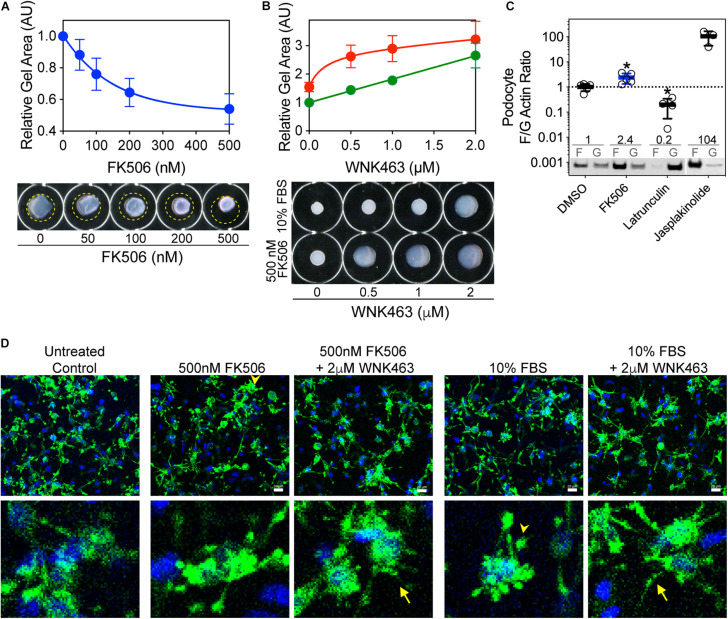
FK506-induced podocyte traction force in floating 3D collagen matrix gels is inhibited by WNK463. **(A)** Effects of increasing concentrations of FK506 on podocyte traction force in 3D collagen matrix. Average gel area ± S.D. of three independent experiments performed in duplicate are shown. The relative areas ± S.D are: C, 1.00; 50 nm, 0.88 ± 0.09; 100 nm, 0.76 ± 0.10; 200 nm, 0.64 ± 0.09; and 500 nm, 0.54 ± 0.1. Representative images of floating collagen matrices are shown, with original matrix size indicated (yellow dotted line). **(B)** Attenuation of FK506- (red) or serum-induced (green) matrix contraction by WNK463. Representative images of floating collagen matrices are shown. **(C)** Podocyte F/G actin ratio is increased by FK506 treatment (0.5 μM) 2.4 ± 0.2-fold, 104 ± 50-fold by Jasplakinolide (1 μm), and decreased to 0.20 ± 14-fold of control by Latrunculin B (1 μm). Values shown are mean ± S.D. and are above representative immunoblots. **(D)** Podocytes in 3D collagen matrix at end of floating matrix contraction assay were fixed and stained for actin to visualize cell extensions. Untreated cells are shown as control. After treatment with 0.5 μM FK506 or 10% FBS, consistent with enhanced contraction measured by reduction in gel size, cells have retracted processes that are in close proximity to the cell bodies (arrowheads). When also treated with 2 μM WNK463, most cell processes remain extended (arrows) with diffuse staining throughout, consistent with decreased retraction force. Insets show higher magnification of cell structures under the five experimental conditions.

An intact podocyte actin cytoskeleton and non-muscle myosins are required for generation of traction force and are basic determinants of the elastic properties of glomerular capillary walls. To assess the effects of FK506 and WNK kinase activity on the ability of podocytes to generate traction force, we used a gel contraction assay which measures the ability of podocytes to bind and move a collagen-based matrix (Figure 4). Cultured podocytes were embedded in 3-D collagen gels, treated with FK506, and their ability to contract the gels was measured (Liu et al., 2014; Embry et al., 2018). FK506 caused a concentration-dependent increase in traction force by the embedded podocytes, as demonstrated by a progressive decrease in gel size (Figure 4A). This effect of FK506 was substantially reduced by WNK463 (Figure 4B). We measured the contribution of WNK activity to gel contraction by 10% serum, which is a strong stimulus for gel contraction (Figure 4B). Gel contraction by both agents was reduced by WNK463; however, higher concentrations of WNK463 were required for slightly less effective blockade of serum-induced gel contraction compared to FK506-induced contraction, suggesting that serum acts in part by a WNK-independent mechanism. Measurements of actin F/G ratios in podocytes after FK506 treatment showed that consistent with enhanced traction force, the F/G actin ratio is increased 2.4-fold over that in control DMSO-treated podocytes (Figure 4C). To investigate the morphologic changes in podocytes and how they relate to gel contraction, podocytes in 3D matrices were imaged with phalloidin staining (Figure 4D). After FBS or FK506-induced gel contraction, extensions are fully retracted with wide, rounded processes, lamellipodia, in the majority of cells. In the gels treated with WNK463 that have reduced traction force, most cells in the 3D matrices have long extensions with a reduced number of the rounded processes seen in the retracted state (Figure 4D; Liu et al., 2014). Supplementary Figure 3 shows higher magnification images of podocytes in gels with FK506, serum, and WNK463 treatment where the effects of the treatments on their structure are more clear. These profound differences in cell morphology after FK506- and serum-stimulated contraction, or with WNK kinase inhibition indicate that WNK activity contributes to actin-mediated cell adhesion, lamellipodium generation, and traction force generation in a collagen matrix.

### WNK Activity Is Necessary for Maintenance of Lamellipodia at Cell Leading Edges

Because calcineurin inhibitors activate WNK and OSR1/SPAK, increase the elastic modulus of glomeruli, the fraction of F-actin, and the traction force of cultured podocytes in 3-D collagen gels, we investigated the effects of FK506 and WNK463 on the morphology and localization of pOSR1/SPAK in cultured podocytes. Brightfield images of cells (panels a, e) show enhanced contrast at membrane protrusions (arrows) that increased in response to FK506. FK506-stimulated membrane edge contrast was reduced by WNK463 (panel i). Confocal images of pOSR1/SPAK (panels b, f, j; green) show punctate cytoplasmic patterns with concentration at the edges of protrusions that is increased by FK506- and reduced in WNK463-treated cells. F-actin/phalloidin staining (panels c, g, k; red) shows a pattern of edge staining with cytoplasmic haziness in untreated cells. In FK506-treated cells, the cell edge staining is thicker and more intense. In the cells treated with FK506 and WNK463, the edge staining is markedly reduced. The merged views (panels d, h, l) show overlap (yellow areas) of pOSR1/SPAK and F-actin staining most easily seen in the high magnification insets. Comparison of the high magnification views of panels d (control) and h (FK506) shows an increase in the width and intensity of actin and pSPAK/OSR1 (yellow) staining indicating increased actin and pSPAK/OSR1 density at the cell edge in cells treated with FK506. Panel l, FK506 + WNK463, shows a marked reduction in the intensity and width of edge staining compared to control or FK506 alone. The effects of FK506 on lamellipodium formation were confirmed by imaging podocytes for cortactin, considered a marker for cortical actin found in lamellipodia, and F-actin (Supplementary Figure 4; Boguslavsky et al., 2007; Sun et al., 2013). An increase in lamellipodia in response to FK506 is readily appreciated as is the effect of WNK463 to reduce lamellipodia (best seen in the high magnification insets).

**FIGURE 5:**
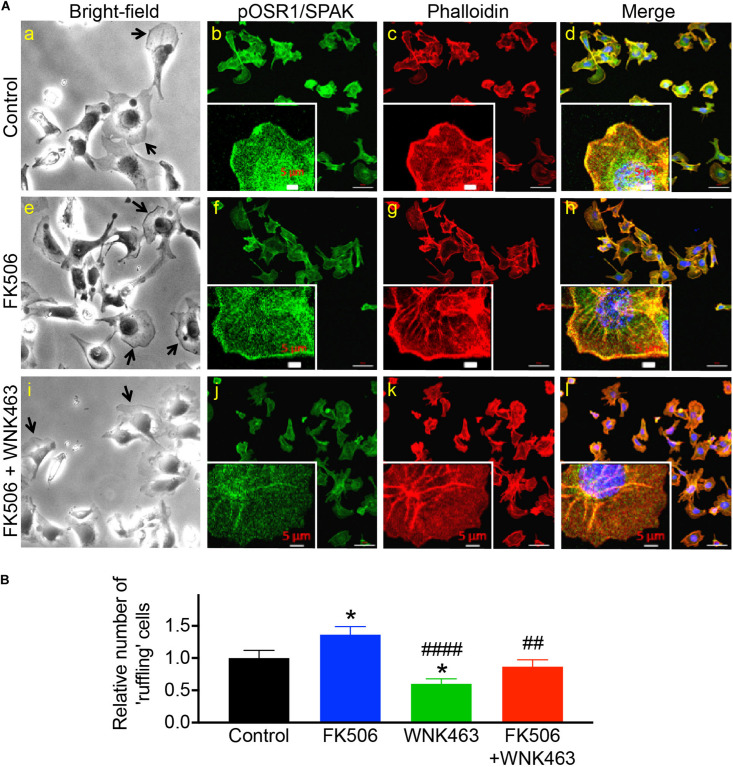
Phospho-OSR1/SPAK co-localization with F-actin at edges of podocyte membrane protrusions is reduced with WNK kinase inhibition. **(A)** Bright-field image of podocytes in culture (panels a, e, i), and confocal microscopy of pOSR1/SPAK (green, panels b, f, j) and F-actin (red, phalloidin panels c, g, k), as indicated. Protruding edges of spread cells in culture are indicated with arrows in bright-field images. Cells treated with FK506 have darker edges in bright-field and confocal images, with visibly stronger co-localization of pOSR1/SPAK and actin (merged panels d, h, l). Localization to the membrane periphery is attenuated with WNK463. **(B)** Relative number of cells with protrusions or “ruffling” cells for control, FK506, WNK463, and FK506 + WNK463 (compared to control) in FBS-containing medium are shown. The values shown are average ± S.E.M from 12 replicate samples and are Control 1.0 ± 0.12, FK506 1.36 ± 0.12, WNK463 0.60 ± 0.08, and FK506 + WNK463, 0.869 ± 0.10; *p < 0.05, compared to control, ^##^*p* < 0.05, and ^####^*p* < 0.001 compared to FK506 by ANOVA.

To quantify changes in pOSR1/SPAK and F-actin in the membrane-edge observed in Figure 5A, the cells were cultured in collagen coated 96 well plates. The average intensity of pOSR1/SPAK in the membrane region was used to determine the number of cells with “ruffles” (see experimental procedure). Under these conditions, FK506 significantly increased the number of cells with membrane ruffling by 136% compared to control, and WNK463 significantly decreased number of cells with ruffling to 60% compared to control. The extent and number of cells with membrane ruffling that were treated with FK506 and WNK463 was similar to that in control (87% of control) and significantly different from FK506 treatment alone (Figure 5B). The effects of FK506 and WNK463 are confirmed in Supplementary Figure 4 with cortactin and F-actin staining.

### FK506 Increases, and WNK Inhibition Decreases Podocyte Migration

**FIGURE 6:**
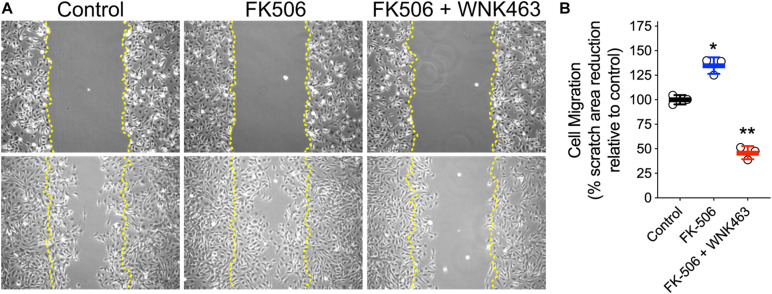
Activation of cell migration by FK506 is attenuated by WNK463-inhibition of WNK kinases. **(A)** Representative images of podocytes at *T* = 0 (top row) and 18 h after indicated treatments (bottom row). Margins of wound from scratched monolayer are shown with yellow dotted lines. **(B)** Cell migration is calculated as percentage original wound area occupied by cells after 18 h in culture in indicated treatment conditions. Cell migration relative to control cells is shown. The control cells migrated to cover 63 ± 3% (Mean ± S.D.) of the denuded area, FK506 treated cells covered 85 ± 5% of the area, but the cells treated with FK506 + WNK463 covered only 29 ± 4% of the area. *N* = 3 independent experiments. *p < 0.05 and **p < 0.01 by ANOVA.

To provide an independent measurement of the functional consequences of increased cell process formation, we used wound healing assays to measure the effects of FK506 and WNK463 on podocyte migration (Figure 6). Images of podocytes immediately after a region of adherent cells was removed (upper row), were compared to images of the same area 18 h after no treatment (Control), FK506 (0.5 μm), or FK506 (0.5 μM) plus WNK463 (2 μM; lower row; Figure 6A). The percentage of the original wound area (outlined in yellow), covered by cells that migrated was quantified. The control cells migrated to cover 60% of the denuded area, FK506 treated cells covered 85% of the area, but the cells treated with FK506 + WNK463 covered only 30% of the area (Figure 6B).

### Depletion of WNK1 Replicates the Effects of WNK463

**FIGURE 7:**
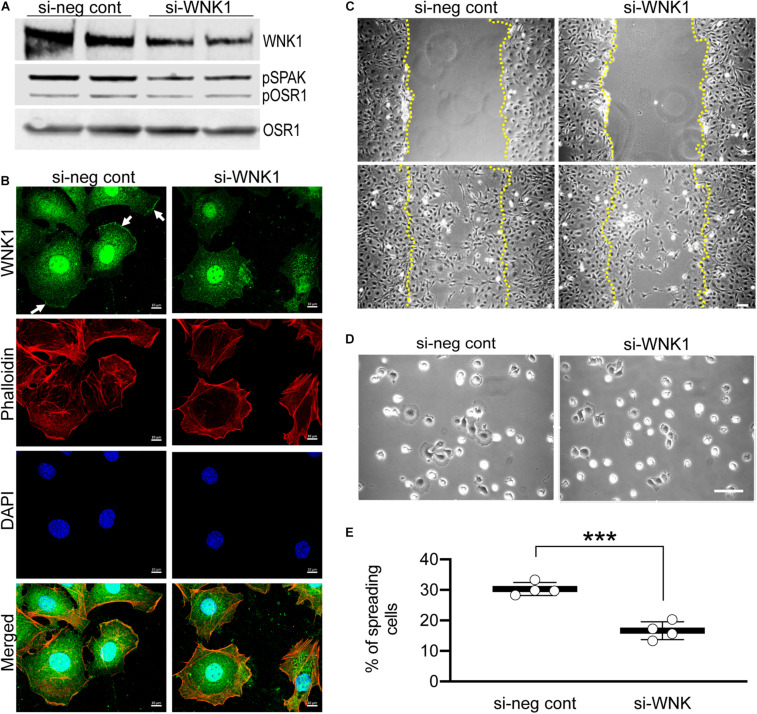
siRNA-mediated depletion of WNK1 replicates the effects of WNK463. Podocytes were transfected with siRNA directed against WNK1 or non-specific control siRNA and studies were carried out 24 h later. **(A)** shows a representative paired experiment comparing the basal level of pOSR1/SPAK in cells transfected with non-specific negative control siRNA (si-neg cont) or the WNK1 targeted siRNA (si-WNK1), as indicated. **(B)** Podocytes transfected with si-neg cont or si-WNK1 were stained with WNK1 antibody (green, enlarged image), rhodamine phalloidin (red, smaller top panel), and DAPI (blue, smaller center panel). Merged (smaller lower panel) image is shown. WNK1 stained panel is enlarged to show localization at the lamellipodia in si-neg cont (arrows) that disappears with WNK1 protein knockdown with si-WNK1. Scale bar = 10 μm. **(C)** Representative images of podocytes transfected with si-neg cont or siWNK1 as indicated at *T* = 0 (upper row) and 22 h (lower row) after treatments. Margins of wound from scratched monolayer are shown with yellow dotted lines. Scale bar = 150 μm. **(D)** Representative images of podocyte cell spreading, captured 2 h after replating, post 48 h after transfection with si-neg cont or si-WNK1. Scale bar = 100 μm. **(E)** Quantification of% of spreading cells in si-neg cont and si-WNK1 transfected cells. The values for si-neg control are 30.3 ± 2.13 and for si-WNK1 are 16.6 ± 2.92, and represent average ± S.D. from 4 random fields of at least 250 cells; ***p < 0.001 by *t*-test.

Because WNK463 is a pan-WNK inhibitor, it cannot distinguish which or how many of the four WNKs mediate the effects we observe. Based on the single cell RNAseq data cited above, we tested WNK1 as the most likely candidate. We depleted WNK1 in podocytes with siRNA achieving a 40–50% reduction in WNK1 protein compared to the control siRNA, that was accompanied by a proportional (∼50%) reduction in pOSR1/SPAK (Figure 7A). If WNK1 is the predominant WNK family member expressed and responsible for lamellipodium formation, cell migration, and cell spreading in cultured cells, the reduction in WNK1 should be accompanied by proportional reductions in these three cell behaviors. In the control (si-neg control) panel stained for WNK1, WNK1 is seen at the leading edges of two cells (arrows) and F-actin is concentrated in the same areas (Figure 7B). Lamellipodium and WNK1 staining at the leading edges of podocytes were reduced in WNK1-depleted podocytes. The two cells on the right side of the si-WNK1 panel stained for WNK1 have irregular borders and less intense staining for WNK1 than the two spread cells on the left of the panel or the cells in the si-neg cont panel and do not have structures that resemble lamellipodia. These cells resemble the cells in Supplementary Figures 1, 2 that were treated with high concentrations of WNK463 and may have the greatest level of WNK1 depletion. The two cells on the left of this panel are spread but do not have structures consistent with lamellipodium formation and may have intermediate levels so WNK1. WNK1 siRNA reduced podocyte migration in the wound healing assay (Figure 7C), as well as the number of spreading cells and lamellipodium formation (Figures 7D,E), compared to that of cells transfected with the control siRNA. WNK1 depletion caused decreases in these WNK463-sensitive responses in proportion to the degree of WNK1 depletion. These results support the conclusion that WNK1 mediates much if not all of the effects of calcineurin inhibitors on cytoskeletal structures in podocytes.

### WNK/OSR1/SPAK Activity Are Required for Structural Integrity of Glomeruli

**FIGURE 8:**
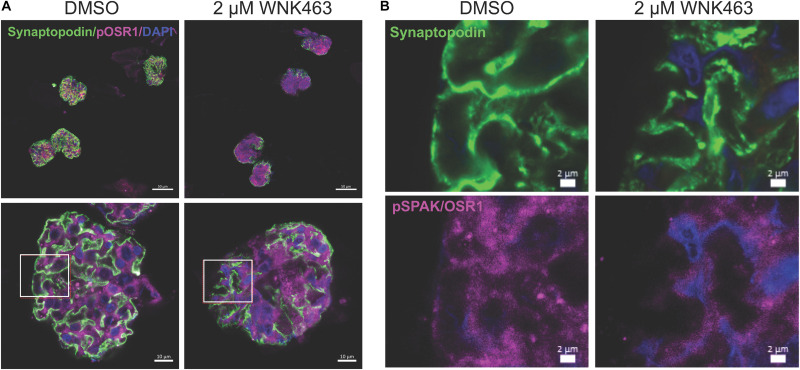
WNK1 and OSR1/SPAK activity are required for normal glomerular capillary structure. Confocal microscopy of isolated mouse glomeruli treated with vehicle (DMSO) or WNK463 (2 μM) for 3 h. Glomeruli were stained for pOSR1/SPAK (magenta), synaptopodin (green), and DAPI (blue) for nuclei. **(A)** The top panels show four glomeruli at low magnification (50 μm scale bars), and the bottom panels show single glomeruli at high magnification (10 μm scale bars). Synaptopodin staining is reduced in the glomeruli treated with WNK463. Insets (white boxes) show the locations of the higher magnification images in **(B)** from the DMSO and WNK463-treated glomeruli (scale bars 2 μm) stained for synaptopdin (green, top), (pOSR1/SPAK magenta, bottom), or nuclei (DAPI, blue). The capillary wall pOSR1/SPAK and synaptopodin staining is even and distinct in the DMSO-treated capillary wall, but not the WNK463-treated capillary wall. In the insets representing the WNK463-treated glomerulus, capillary wall structure is disrupted with reduced intensity of pOSR1/SPAK staining and absence in regions of synaptopodin staining. The synaptopodin staining is irregular in contour and intensity.

*In vivo* cells function in a highly complex three-dimensional microenvironment, composed of multiple matrix proteins, direct contact and communication with other cells, and soluble factors. They cannot develop comparable cell morphology or structure within a less complex *in vitro* environment. Extending our biophysical measurements of glomerular stiffness and podocyte cell contraction and migration, we used confocal microscopy of freshly isolated glomeruli to determine if WNK kinase activity contributes to podocyte and glomerular capillary structure (Figure 8). Staining for synaptopodin (green), pOSR1/SPAK (magenta), and nuclei (blue, DAPI) in DMSO (vehicle control), or WNK463 treated glomeruli shows that inhibition of WNK activity caused dramatic structural changes in the podocytes, resulting in a fundamentally different appearance of glomeruli (Figure 8A). The DMSO-treated glomeruli have the expected structure and patterns of staining for synaptopodin and pOSR1/SPAK can be seen along capillary walls. In contrast, treatment with 2 μM WNK463 leads to dramatically reduced and irregular synaptopodin and pOSR1/SPAK staining (Figure 8B). Unlike the appearance in control cells, pOSR1/SPAK staining in capillary walls in a pattern that corresponds to synaptopodin staining is markedly reduced. These images suggest dramatic changes in cell structure and cell-matrix interactions with WNK inhibition.

**FIGURE 9:**
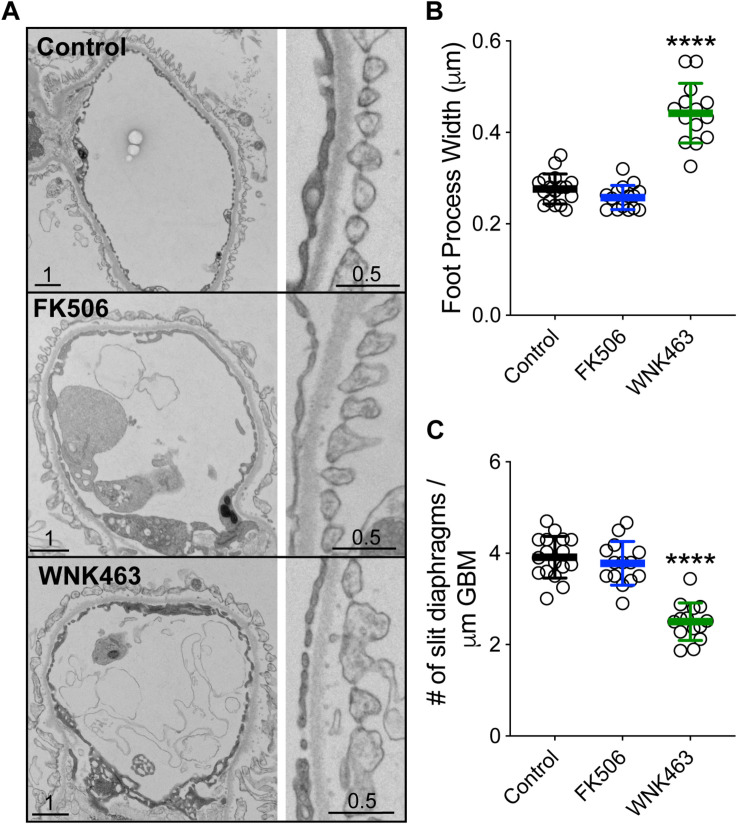
WNK1 activity controls glomerular capillary and podocyte foot process structure. **(A)** Isolated mouse glomeruli were treated with DMSO (Control, top panel), FK506 (500 μM, middle panel), or WNK463 (2 μM bottom panel) for 2 h and fixed, **(B)** Summary of podocyte foot process width measurements. The foot processes are approximately μm wide [0.28 ± 0.04 μm (Control, *n* = 17) and 0.26 ± 0.06 μm FK506, *n* = 17]. Treatment with 2 mM WNK463 for 2 h resulted in wider and irregularly-sized foot processes **(B)**. In these samples, the foot processes are 0.478 ± 0.647 μm (*n* = 15). **(C)** Summary of slit diaphragm density measurements. The slit diaphragm density was similar in the control and FK506-treated mice, 3.91 ± 0.44 and 4.49 ± 1.05 SD/μm GBM, respectively. **(B)** Slit diaphragm density was reduced from 3.91 ± 0.44 (control) to 2.41 ± 0.5 (number SD/μm GBM) by WNK463 treatment. Isolated glomeruli were fixed in glutaraldehyde and cacodylate buffer, and processed as described in Methods. Images shown were obtained at 3,000×. In each condition, the widths of approximately 200 foot processes over approximately 200 μm of GBM were measured from at least six separate glomeruli. Values shown are mean ± SD. ****p < 0.0001 by ANOVA with multiple comparisons post-test against Control.

In order to define the effects of FK506 and WNK643 on glomerular capillaries at the ultrastructural level, we used transmission electron microscopy of isolated glomeruli treated for 2 h with vehicle (DMSO), FK506 (0.5 μm), or WNK463 (2.0 μm) to analyze podocyte and foot process structure (Pisarek-Horowitz et al., 2020). Transmission electron microscopy showed that FK506 has a minimal effect on the structure, size, or organization of foot processes (Figures 9A–C). The foot processes are approximately 3 μm wide (0.28 Control, 0.26 FK506), and the slit diaphragm density was similar in the control and FK506-treated mice, 4–4.4 SD/μm GBM (Figures 9B,C). However, treatment with WNK463 resulted in wider and irregularly-sized foot processes, demonstrating early signs of podocyte injury (Figure 9A). In these samples, the foot processes were wider, approximately 0.5 μm on average (Figure 9B), and the slit diaphragm density was reduced by approximately 40% from 3.9 (control) to 2.4 slit diaphragms/μm GBM (Figure 9C).

## Discussion

The podocyte actin cytoskeleton is essential for the normal functions of podocytes including their interactions with matrix and neighboring podocytes. These matrix and cell-cell interactions determine the ability of podocytes to adhere to the glomerulus, as well as the mechanical properties of podocytes and glomerular capillaries. Following experimental injury and in renal disease, podocytes are lost from glomeruli at an increased rate leading to cycles of hypertrophy and further podocyte loss (Wiggins et al., 2005; Faul et al., 2008; Ding et al., 2017; Lemley, 2017; Nishizono et al., 2017; Schell and Huber, 2017).

In small clinical studies and collections of patient cases with kidney diseases including WT1 mutation-associated glomerular disease, membranous nephropathy, and Alport nephropathy, CsA or FK506 can improve the course of the kidney disease (Charbit et al., 2007; Waldman et al., 2007; Bensman and Niaudet, 2010; Chiba and Inoue, 2019). Alport nephropathy, for example, is caused by mutations in type IV collagen which forms the GBM; calcineurin inhibitor-based improvement in this disease occurs by mechanisms that appear to be independent of T-cell involvement (Charbit et al., 2007; Bensman and Niaudet, 2010). Consistent with effects observed in patients, calcineurin inhibitors preserve podocyte cytoskeletal structure and glomerular integrity in mouse experimental models following exposure to lipopolysaccharide, puromycin aminonucleoside, or protamine sulfate, also by mechanisms that are T cell-independent. Proposed mechanisms for preservation of podocyte structure include prevention of synaptopodin degradation, reduction in injury-induced WAVE1 [Wiscott-Aldrich Syndrome Protein (WASP)-family protein 1] expression, and inhibition of apoptosis (Faul et al., 2008; Vassiliadis et al., 2011; Li et al., 2015; Shen et al., 2016; Buvall et al., 2017). Because calcineurin inhibitors activate WNKs, we investigated the contribution of WNK regulation by calcineurin inhibitors to the calcineurin inhibitor-mediated improvement in glomerular podocyte function and glomerular structure.

Using a distinct set of assays for glomerular capillary and podocyte cell structure and function, we found evidence for a mechanism involving calcineurin, WNK kinases and OSR1/SPAK kinases that regulates podocyte cytoskeleton structure, foot process structure, and glomerular capillary wall structure and elasticity. Stabilization of podocytes on the GBM and glomeruli with increased capillary stiffness mediated by the calcineurin-WNK-OSR1/SPAK signaling pathway-stimulated increase in F-actin may contribute to protection of podocytes and glomeruli in some glomerular diseases and disease models where a component of glomerular injury is softening of glomeruli and podocytes (Wyss et al., 2011; Embry et al., 2016, 2018; Calizo et al., 2019; Ge et al., 2020). These discoveries in renal glomeruli may be applicable to other cells and tissues where WNKs may have a fundamental role in regulating cytoskeleton, cell, and tissue structure, and mechanical properties.

Several lines of evidence suggest that WNK1 is the primary mediator of these processes. The high degree of WNK463 specificity for WNK family members and involvement of OSR1/SPAK in these events clearly implicate WNK kinases as the integrators of these podocyte and glomerular capillary functions (Zhang et al., 2016; Yamada et al., 2016a, b). WNK1 is expressed ubiquitously and has a central role in numerous cell behaviors notably in endothelial cells where WNK1 knockdown has effects similar to those of WNK463 in podocytes and on glomerular cytoskeletal structure (Xie et al., 2009, 2013; Dbouk et al., 2014). We find that WNK1 protein is expressed in cultured podocytes and glomeruli in a pattern consistent with podocyte expression. Expression of the other WNK kinases in the glomerulus has not been unequivocally defined, but snRNAseq data from the Humphreys laboratory at Washington University (see text footnote 1) shows that WNK1 mRNA is the predominant form expressed in glomerular cells, particularly podocytes, suggesting minimal contributions of other family members.

With No Lysine 1 has been implicated in cytoskeletal structure and cell motility for more than 10 years but mechanistic details have emerged only recently. WNK463 inhibits corneal wound healing and reduces filopodium formation in engineered corneas and cultured corneal epithelial cells where WNK1 is the predominant WNK isoform expressed (Desjardins et al., 2019). A pathway including WNK1, SPAK and NKCC1 mediates vascular tone and the pressor response to α_1_ adrenergic agonists in WNK1^+/−^ mice (Bergaya et al., 2012). Knockdown of WNK1 reduces cord formation and migration toward serum by human vein endothelial cells (Dbouk et al., 2014). In T lymphocytes, activation of WNK1 by the T-cell receptor or CCR7 decreases T-cell adhesion to LFA and increases migration, both by WNK1 kinase-dependent mechanisms. Stimulation of migration in T lymphocytes also requires activation of OSR1/SPAK and NKCC1 (SLC12A2), and is convergent with models that involve Cl flux-determination of cell volumes at the cell leading and training edges (Kochl et al., 2016). WNK1 and 3 and their regulation of OSR1/SPAK and NKCC1 are essential for glioblastoma cell migration (Haas et al., 2011; Zhu et al., 2014). This mechanism may be applicable to podocytes and glomeruli, where we identified WNK1 and OSR1/SPAK activity-dependent changes in cell membrane morphology and migration as well as capillary elasticity.

We found that calcineurin inhibitors increased OSR1/SPAK activity in cultured podocytes, in glomerular homogenates, and in capillary walls, and led to increased levels of F-actin, lamellipodium formation with increased cortical actin density, cell migration in monolayers, increased stress fibers, and podocyte traction force in 3-D collagen gels. All effects were sensitive to WNK inhibition. Additionally, in intact isolated glomeruli, a native 3-D structure with intact podocyte geometry and cell interactions, activation of WNK/OSR1/SPAK stiffens glomeruli. This response is at least partly attributable to increased cortical F-actin in podocytes, and may also reflect other alterations in cell structure, such as cell-cell adhesion, cell-matrix adhesion, and possibly cell volume (Kochl et al., 2016; Embry et al., 2016, 2018). Treatment of glomeruli with a WNK inhibitor resulted in loss of active OSR1/SPAK in capillary walls, progressive loss of synaptopodin staining, disruption of capillary wall structure, and softening of capillary walls. Transmission electron microscopy images of glomeruli after acute WNK inhibition *ex vivo*, shows widening of foot processes and a reduction in slit diaphragm density consistent with early podocyte injury. The findings that WNK463 causes specific structural changes in cultured podocytes (loss of lamellipodia, reduced cortical actin) that are reversible with drug removal, and that WNK kinase inhibition disrupts glomerular capillary structure with widening of foot processes and reduction in slit diaphragm density are evidence for specific WNK effects on cell and tissue structure in 2-D and 3-D environments, and not toxicity (Supplementary Figures 3, 4).

Some studies in 2-D culture systems associate increased podocyte motility and lamellipodium formation with podocyte injury and glomerular pathology (Asanuma et al., 2006; Akilesh et al., 2011). We find that WNK1 contributes to increased podocyte motility and lamellipodium formation in 2-D culture systems in concert with increased F-actin formation. In 3-D systems, WNK1 activation is associated with increased podocyte contractility and increased stiffness of glomerular capillary walls, concurrent with increased F-actin formation. These effects are consistent with the protective effects of calcineurin inhibitors on podocytes and glomeruli in disease models and some human diseases where evidence exists for podocyte and capillary softening as part of the disease process (Wyss et al., 2011; Embry et al., 2016, 2018; Calizo et al., 2019; Ge et al., 2020).

In 2-D and 3-D environments, cell migration utilizes the same basic biochemical mechanisms including small GTPase-regulated actin cytoskeletal rearrangements, but the processes by which the cells move may differ (Asanuma et al., 2006; Akilesh et al., 2011; Paluch et al., 2016; Yamada and Sixt, 2019). In 2-D environments, cells migrate by extending lamellipodia or sometimes filopodia in the direction of travel, forming adhesions to matrix, and retracting the rear of the cell. In 3-D environments, where cells are more confined, migration is thought to occur through “mesenchymal” or “ameboid” mechanisms, or a combination of the two (Paluch et al., 2016). Mesenchymal migration in 3-D is characterized by extension of lamellipodia or filopodia-like structures into the matrix, and establishment of adhesive contacts followed by retraction of the cell in the direction of migration, similar to migration in 2-D environments. Ameboid migration is characterized by extensive changes in cell shape without specific interactions with the matrix. The structures involved in ameboid motility are blebs, microscopic cytoplasm-filled bulges in the plasma membrane, and lobopodia, bleb-like protrusions at the leading edge driven by cytoskeleton-generated asymmetric hydrostatic pressure gradients within the cell. Lobopodia may result from changes in local cytoskeletal rearrangement and generalized or localized cell volume alterations (Paluch et al., 2016). Our results indicate that WNK1 signaling is important for migration and by implication regulated changes in cytoskeletal structure in both 2-D and 3-D environments.

In conclusion, we demonstrated that WNK1 and OSR1/SPAK are essential for maintenance of normal glomerular and podocyte architecture and biomechanical properties. WNK1 and its substrate kinases OSR1/SPAK are expressed in isolated glomeruli, glomerular podocytes, and cultured podocytes with similar cytoplasmic distributions. Our studies reveal that WNK1 and OSR1/SPAK kinases regulate actin structures and affect cell interactions with matrix and neighboring cells. We found that FK506 and CsA activate WNK1 and OSR1/SPAK in these cells and their activation causes an increase in F-actin that leads to increased stiffness of glomeruli and increased cell protrusions in cultured podocytes. These structural changes manifest as cell migration in 2D culture or collagen gel contraction in 3-D culture. Our work reveals new functions for WNK and OSR1/SPAK kinases, and suggests an important mechanism by which podocyte cytoskeletal structure and matrix interactions can be modified, and by which calcineurin inhibitors may protect podocytes and glomeruli from injury.

## Data Availability Statement

The raw data supporting the conclusions of this article will be made available by the authors, without undue reservation.

## Ethics Statement

The animal study was reviewed and approved by UT Southwestern Medical Center Animal IACUC.

## Author Contributions

RTM, MHC, ZL, ANC, and CW: conceptualization and formal analysis. ZL, JY, AE, LL, HAD, and ABJ: investigation. ZL, JY, JMH, MHC, and RTM: methodology. RTM, ANC, JMH, MHC, and ZL: writing first draft, revisions, and editing. ANC, RTM, and MHC: supervision. RTM and MHC: funding Acquisition. All authors contributed to the article and approved the submitted version.

## Conflict of Interest

The authors declare that the research was conducted in the absence of any commercial or financial relationships that could be construed as a potential conflict of interest.

## Footnotes

1 http://humphreyslab.com/SingleCell/
