# Supplementary material for: Control of Podocyte and Glomerular Capillary Wall Structure and Elasticity by WNK1 Kinase
Source: Front Cell Dev Biol. 2021 Feb 2;8:618898. doi: 10.3389/fcell.2020.618898 (PMC7884762; doi:10.3389/fcell.2020.618898)
Supplement: Supplementary file 1 [file Presentation_1.pdf]

Supplementary Data

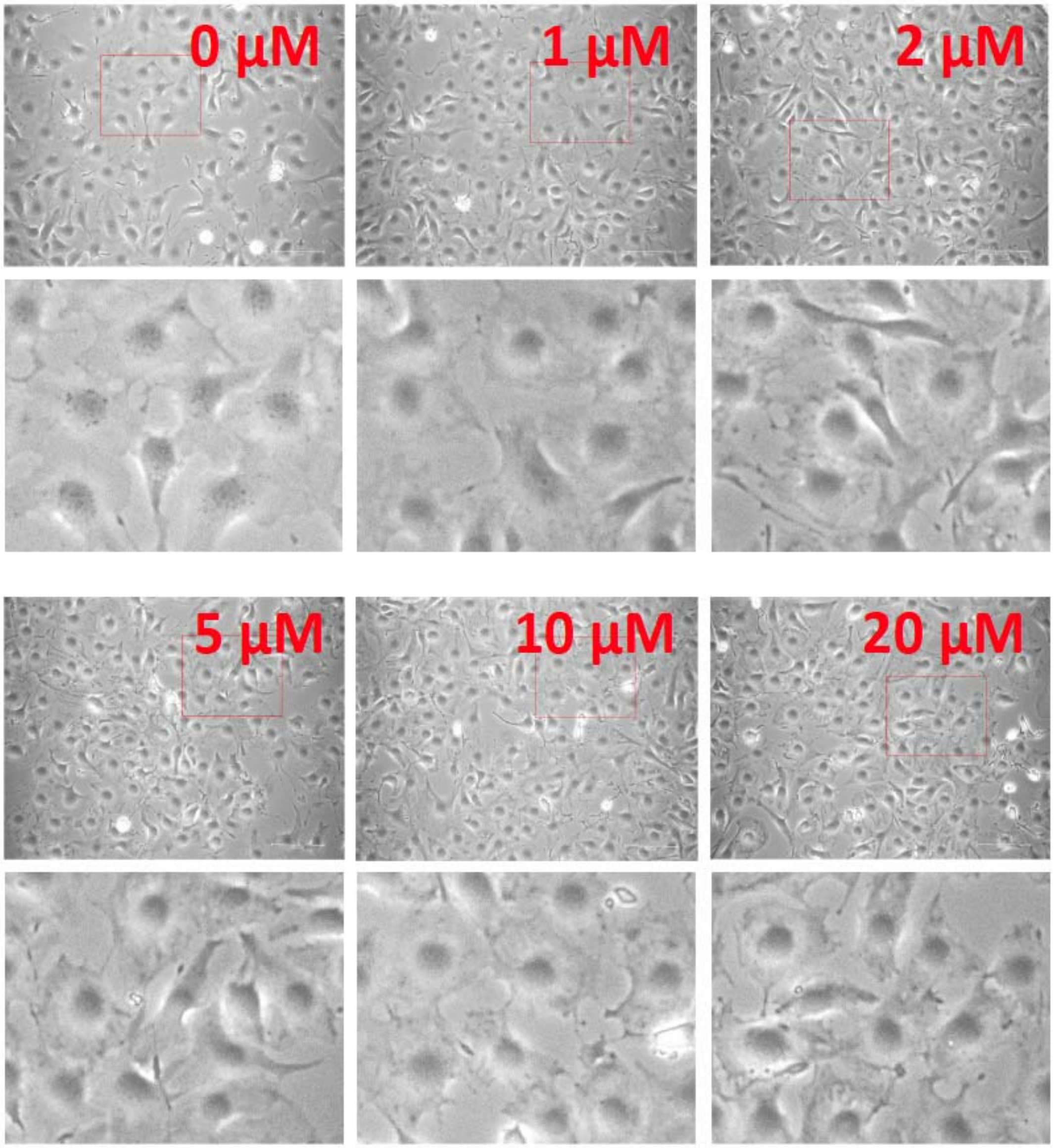

Figure S1. Dose-effect of WNK463 on podocyte cell adhesion and morphology in standard culture conditions. Podocytes were cultured in a 6-well dish in FBS-supplemented culture medium. To determine whether high doses of WNK463 were toxic for the podocytes in culture, increasing concentrations of WNK463 were added to each well for three hours as indicated. Bright-field images were taken at low magnification to compare cell density, and a selected region enlarged to evaluate cell morphology. Even at the highest WNK463 concentration of 20 µM, cells were adherent and appeared healthy.

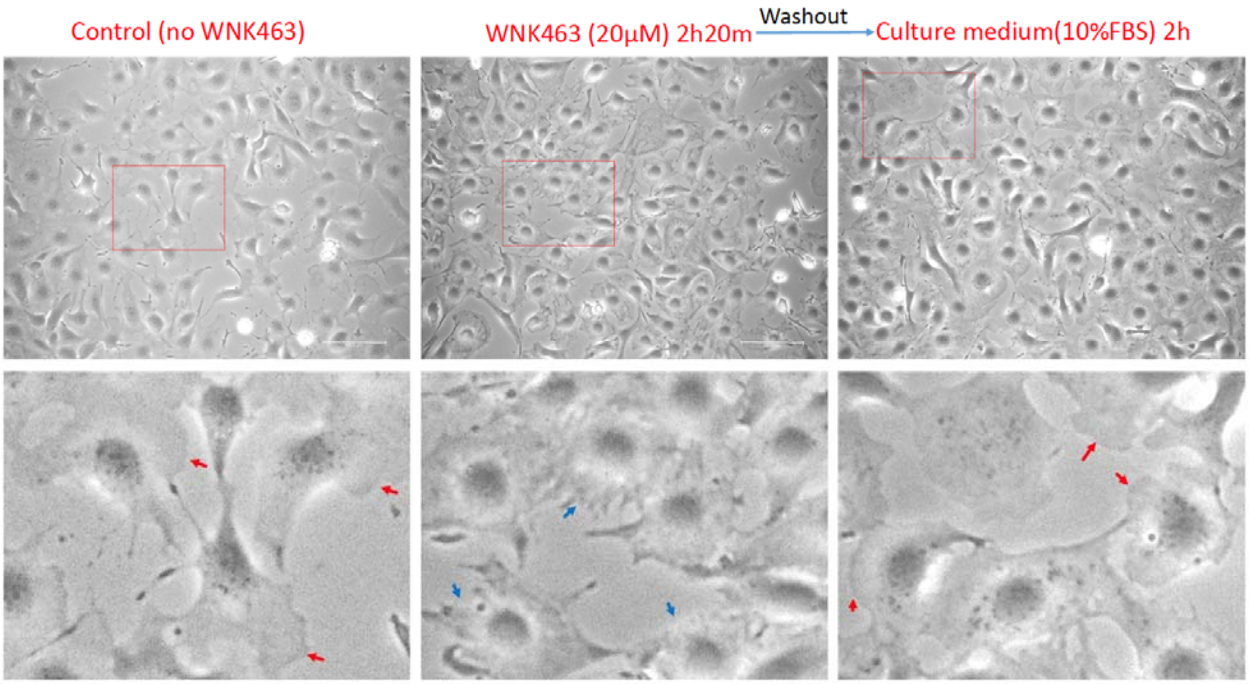

Figure S2. Evaluation of reversibility of WNK463-induced changes in podocyte cell morphology. Podocytes were cultured in a 6-well dish in FBS-supplemented culture medium. The effects of a high concentration of WNK463 (20 μM, three h) on cell membrane morphology was imaged by bright-field microscopy. Absence of lamellipodia (red arrows) in the presence of 20 μM WNK463 and enrichment of filopodia-like structures (blue arrows) is illustrated. After washout of WNK463, the majority of the cells reverted to untreated control morphology.

A.

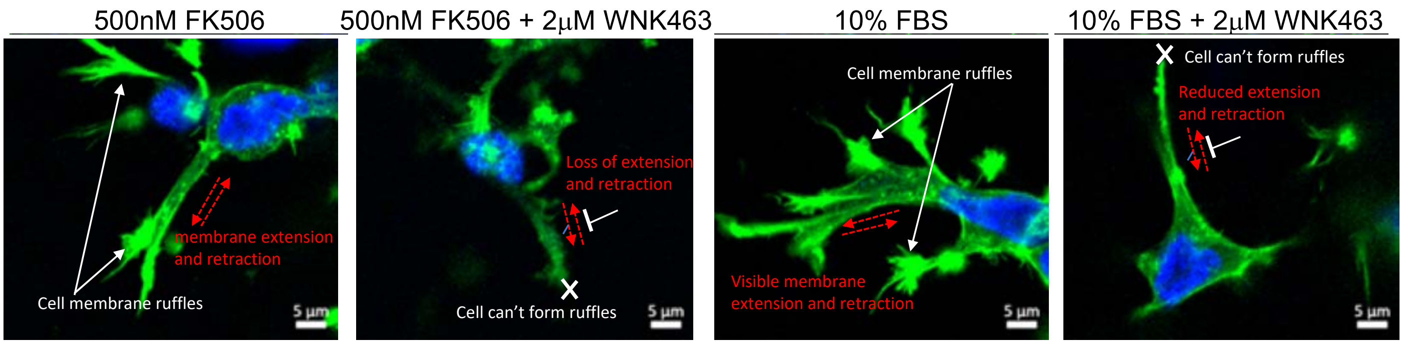

B.

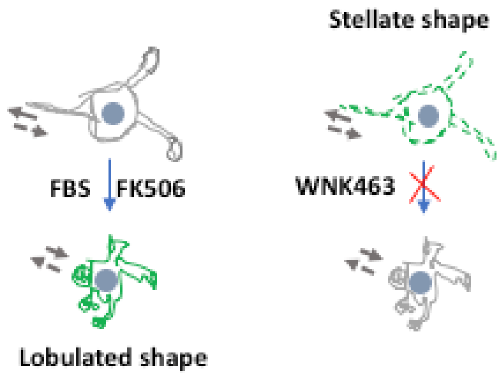

Figure S3. Comparison of podocyte cell morphology at high magnification. A) Podocytes in 3D gels treated with FBS or FK506, in the absence or presence of WNK463 co-treatment as indicated. **B)** Schematic of podocyte membrane extensions, retraction and ruffles inside 3D Collagen I matrices. In the absence of WNK463 (-), FBS or FK506 both induce formation of membrane extensions with short, rounded membrane protrusions. Cell membrane extensions are retracted and membrane ruffles (lobular protrusions) are increased, resulting in matrix remodeling and contraction. In the presence of WNK463 (+), WNKs are inhibited, and cells have notably thinner, rod-like protrusions. Rod-like stellate-shaped cells indicate formation of cell membrane dynamic extensions, but without lamellipodia (ruffling), retraction, and cell-matrix remodeling.

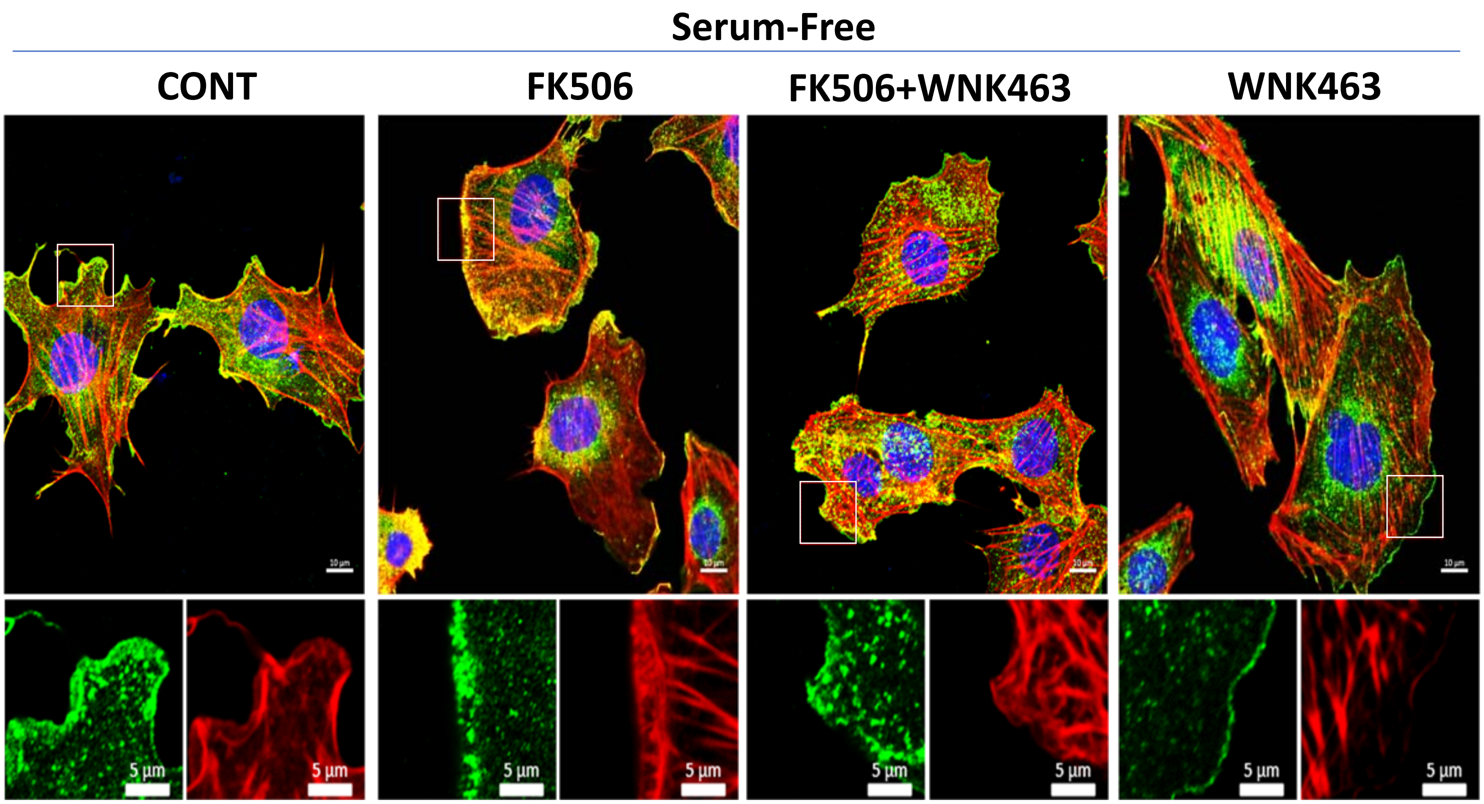

Figure S4. Definition of lamellipodia with Cortactin staining. Podocytes were plated on collagen I-coated coverslips in culture. Cells were washed 3 times with serum-free medium after overnight attachment and spreading, then treated with DMSO (cont), FK506 (0.5μM), or FK506+WNK463, and WNK463 (1μM) for 3h. After fixation, cells were stained with the cortactin antibody (1:100) overnight at 4°C, followed by 1h incubation with Alex 488-goat anti-rabbit (1:500) and rhodamine phalloidin (1:1000), and then mounted on glass slides using mounting media with DAPI for nuclear staining. Cortactin (green), phalloidin stained F-actin (red), and DAPI stained nuclei (blue), are shown. Scale bar: 10 μm on upper merged images, 5 μm on lower split magnified images that show cortactin (green) and F-actin (red).
